# Supplementary material for: In vitro characterization and in vivo comparison of the pulmonary outcomes of Poractant alfa and Calsurf in ventilated preterm rabbits
Source: PLoS One. 2020 Mar 13;15(3):e0230229. doi: 10.1371/journal.pone.0230229 (PMC7069639; doi:10.1371/journal.pone.0230229)
Supplement: S1 Method — (DOCX) [file pone.0230229.s001.docx]

**S1 Method**

**Liquid-Chromatography Mass-Spectrometry method for phospholipids analysis**

Liquid-Chromatography solvents, acetic acid and ammonia solution were purchased from Sigma Aldrich Italy (Milan, Italy) and were of the best analytic grade available. The chemical standards of phospholipids (PLs) and the internal standards used for quantitative purposes (at least one representative molecule for each class of PLs measured) were obtained from Avanti Polar Lipids (Alabaster, AL, USA) and were: 1-palmitoyl-2-hydroxy-*sn*-glycero-3-phosphocholine, 1,2-dipalmitoyl-*sn*-glycero-3-phosphocholine (DPPC), 1-palmitoyl-2-arachidonoyl-*sn*-glycero-3-phosphocholine, N-palmitoyl-D-*erythro*-sphyngosylphosphoryl-choline, 1-stearoyl-2-oleoyl-*sn*-glycero-3-phosphoethanolamine, 1-O-1’-octadecenyl-2-arachidonoyl-*sn*-glycero-3-phosphoethanolamine, 1,2-dioleoyl-*sn*-glycero-3-phosphoinositol, 1-palmitoyl-2-oleoyl-*sn*-glycero-3-phospho-*rac*-(1-glycerol), 1-heptadecanoyl-2-(9-tetradecenoyl)-*sn*-glycero-3-phosphoglycerol, 1-heptadecanoyl-2-(5,8,11,14-eicosatetraenoyl)-*sn*-glycero-3-phosphoglycerol, 1-heptadecanoyl-2-(9-tetradecenoyl)-*sn*-glycero-3-phosphocholine, 1-(10-heptadecenoyl)-2-hydroxy-*sn*-glycero-3-phosphocholine, N-(dodecanoyl)-sphing-4-enine-1-phosphocholine, 1-heptadecanoyl-2-(9-tetradecenoyl)-*sn*-glycero-3-phosphoethanolamine, and 1-heptadecanoyl-2-(9-tetradecenoyl)-*sn*-glycero-3-phosphoinositol. The chemical standards of cholesterol and free fatty acids (FFAs) were from Sigma Aldrich; they were palmitic acid, oleic acid, arachidonic acid, stearic acid, myristic acid, and linoleic acid. The 10-heptadecenoic acid was used as the internal standard for FFAs.

Normal Phase Liquid Chromatography-Mass Spectrometry (NPLC-MS) was performed on a Thermo Scientific (Bremen, Germany) system composed by a HPLC Surveyor, with column oven and autosampler, coupled to a LTQ ion trap mass spectrometer via electrospray (ESI) interface (Thermo). Surfactant samples were separated on a Varian Polaris Si A, 250 x 2.1 mm, 5 µm, 200 Å (Agilent Technologies, Santa Clara, CA, USA) with a ternary gradient, using the following eluents:

eluent A: CHCl_3_:MeOH:NH_3_: Acetic Acid (AcAc) 58.7:40.0:1.0:0.3 (v:v:v:v)

eluent B: CHCl_3_:MeOH:NH_3_ 84.0:15.0:1.0 (v:v:v)

eluent C: CHCl_3_:MeOH:H_2_O:NH_3_:AcAc 60.0:34.0:4.7:1.0:0.3 (v:v:v:v:v)

and gradient program:

| Time (min) | A (%) | B (%) | C (%) |
| --- | --- | --- | --- |
| 0.0 | 0 | 100 | 0 |
| 0.5 | 0 | 100 | 0 |
| 12.5 | 60 | 40 | 0 |
| 14.0 | 0 | 0 | 100 |
| 19.0 | 0 | 0 | 100 |
| 20.0 | 0 | 100 | 0 |

A multistep LC gradient was employed with 100% eluent B that turned to 100% eluent C over 19 min and finally, the starting gradient was restored in 1 min and maintained for 15 min for column reconditioning. Column temperature was set at 45 °C and flow rate at 0.35 mL/min. Gradient elution was obtained over 20 min, then the column was reconditioned for 15 min in the starting condition. Acquisition was performed by polarity switching, recording both negative and positive ions during the entire elution program. Data were acquired in full scan mode, from 200 to 1600 m/z, excluding cholesterol that was acquired in positive ion MS/MS mode, recording the two product ions at m/z 161.1 and 243.2 deriving from the precursor ion at m/z 369.3, [M+H-H_2_O]^+^ (isolation window 2.6 m/z, normalized collision energy 20). The ESI interface and ion optics main parameters were reported in the following table:

| Positive ions | Parameter | Negative ions |
| --- | --- | --- |
| 4100 | Spray voltage (V) | -3600 |
| 44 | Sheath gas flow (arbitrary unites) | 44 |
| 6 | Sweep gas flow (arb. units) | 6 |
| 4 | Auxiliary gas flow (arb. units) | 4 |
| 295 | Capillary temperature (^o^ C) | 295 |
| 20.3 | Capillary voltage (V) | -19 |
| 65 | Tube lens offset (V) | -70 |

Data were acquired and elaborated by Xcalibur software version 2.0.7. Semi-quantitative data were accessed by manual peak integration of the analytes of interest.
