# Supplementary material for: In vitro characterization and in vivo comparison of the pulmonary outcomes of Poractant alfa and Calsurf in ventilated preterm rabbits
Source: PLoS One. 2020 Mar 13;15(3):e0230229. doi: 10.1371/journal.pone.0230229 (PMC7069639; doi:10.1371/journal.pone.0230229)
Supplement: S1 Table — Values are mean+SD, or n (%). Group definitions: P200, Poractant alfa 200 mg/kg; P100, Poractant alfa 100 mg/kg; C200, Calsurf 200 mg/kg; C100, Calsurf 100 mg/kg; C70, Calsurf 70 mg/kg; Ctrl, control. ** P<0.01 and * P<0.05 vs. Ctrl group by Chi square test. Initial numbers = 25 in each group. (DOCX) [file pone.0230229.s002.docx]

**S1 Table.**

Birth weight (BW) and survival rate of preterm rabbits assessed for 180 min standardized V_T_ mechanical ventilation.

| Groups | BW (g) | Survival number (%) over time (min) | | | | | | |
| --- | --- | --- | --- | --- | --- | --- | --- | --- |
|  |  | 15 | 30 | 45 | 60 | 90 | 120 | 180 |
| Ctrl | 27.5+5.0 | 23 (92) | 21 (84) | 16 (64) | 15 (60) | 13 (52) | 12 (48) | 10 (40) |
| P200 | 26.8+5.3 | 25 (100) | 25 (100) | 24 (96)** | 24 (96)** | 22 (88)** | 21 (84)** | 19 (76)* |
| P100 | 26.6+4.3 | 25 (100) | 24 (96) | 23 (92) | 20 (80) | 18 (72) | 18 (72) | 15 (60) |
| C200 | 27.1+4.3 | 25 (100) | 24 (96) | 23 (92)* | 21 (84) | 19 (76) | 17 (68) | 14 (56) |
| C100 | 27.0+3.9 | 25 (100) | 24 (96) | 21 (84) | 18 (72) | 17 (56) | 16 (52) | 12 (48) |
| C70 | 26.7+3.6 | 24 (96) | 23 (92) | 17 (68) | 16 (64) | 14 (56) | 13 (52) | 10 (40) |

Values are mean+SD, or n (%). Group definitions: P200, *Poractant alfa* 200 mg/kg; P100, *Poractant alfa* 100 mg/kg; C200, *Calsurf* 200 mg/kg; C100, *Calsurf* 100 mg/kg; C70, *Calsurf* 70 mg/kg; Ctrl, control. ** *P*<0.01 and * *P*<0.05 vs. Ctrl group by Chi square test. Initial numbers =25 in each group.
