# Supplementary material for: In vitro characterization and in vivo comparison of the pulmonary outcomes of Poractant alfa and Calsurf in ventilated preterm rabbits
Source: PLoS One. 2020 Mar 13;15(3):e0230229. doi: 10.1371/journal.pone.0230229 (PMC7069639; doi:10.1371/journal.pone.0230229)
Supplement: S2 Table — Values are mean+SD. a: p values are for one-way analysis of variance (ANOVA) by F test; * p<0.05 for between-group differences between Ctrl and any surfactant-treated groups by Student-Newmann-Keuks post hoc test. For group definitions and sample sizes at each time point see S1 Table in the Supporting information. (DOCX) [file pone.0230229.s003.docx]

**S2 Table.**

Dynamic compliance of respiratory system (C_dyn_) over time in preterm rabbits with prophylactic surfactant treatment and standardized V_T_ mechanical ventilation.

| Groups | Ventilation time (min) | | | | | | |
| --- | --- | --- | --- | --- | --- | --- | --- |
|  | 15 | 30 | 45 | 60 | 90 | 120 | 180 |
| Ctrl | 0.14+0.06* | 0.15+0.07* | 0.17+0.09* | 0.15+0.10* | 0.14+0.08* | 0.14+0.08* | 0.12+0.08* |
| P200 | 0.28+0.10 | 0.30+0.11 | 0.30+0.11 | 0.29+0.08 | 0.34+0.12 | 0.31+0.13 | 0.32+0.12 |
| P100 | 0.29+0.18 | 0.29+0.13 | 0.30+0.15 | 0.29+0.14 | 0.33+0.12 | 0.32+0.12 | 0.31+0.12 |
| C200 | 0.27+0.11 | 0.26+0.09 | 0.29+0.13 | 0.29+0.09 | 0.28+0.10 | 0.29+0.08 | 0.28+0.10 |
| C100 | 0.23+0.10 | 0.27+0.09 | 0.31+0.10 | 0.29+0.12 | 0.28+0.17 | 0.28+0.15 | 0.26+0.17 |
| C70 | 0.22+0.14 | 0.23+0.12 | 0.25+0.16 | 0.26+0.15 | 0.26+0.16 | 0.28+0.14 | 0.25+0.10 |
| *P* ^a^ | 0.000 | 0.001 | 0.024 | 0.001 | 0.001 | 0.001 | 0.001 |

Values are mean+SD. a: *p* values are for one-way analysis of variance (ANOVA) by F test; * *p*<0.05 for between-group differences between Ctrl and any surfactant-treated groups by Student-Newmann-Keuks post hoc test. For group definitions and sample sizes at each time point see S1 Table in the Supporting information.
