# Supplementary figures and images for: In vitro characterization and in vivo comparison of the pulmonary outcomes of Poractant alfa and Calsurf in ventilated preterm rabbits
Source: PLoS One. 2020 Mar 13;15(3):e0230229. doi: 10.1371/journal.pone.0230229 (PMC7069639; doi:10.1371/journal.pone.0230229)

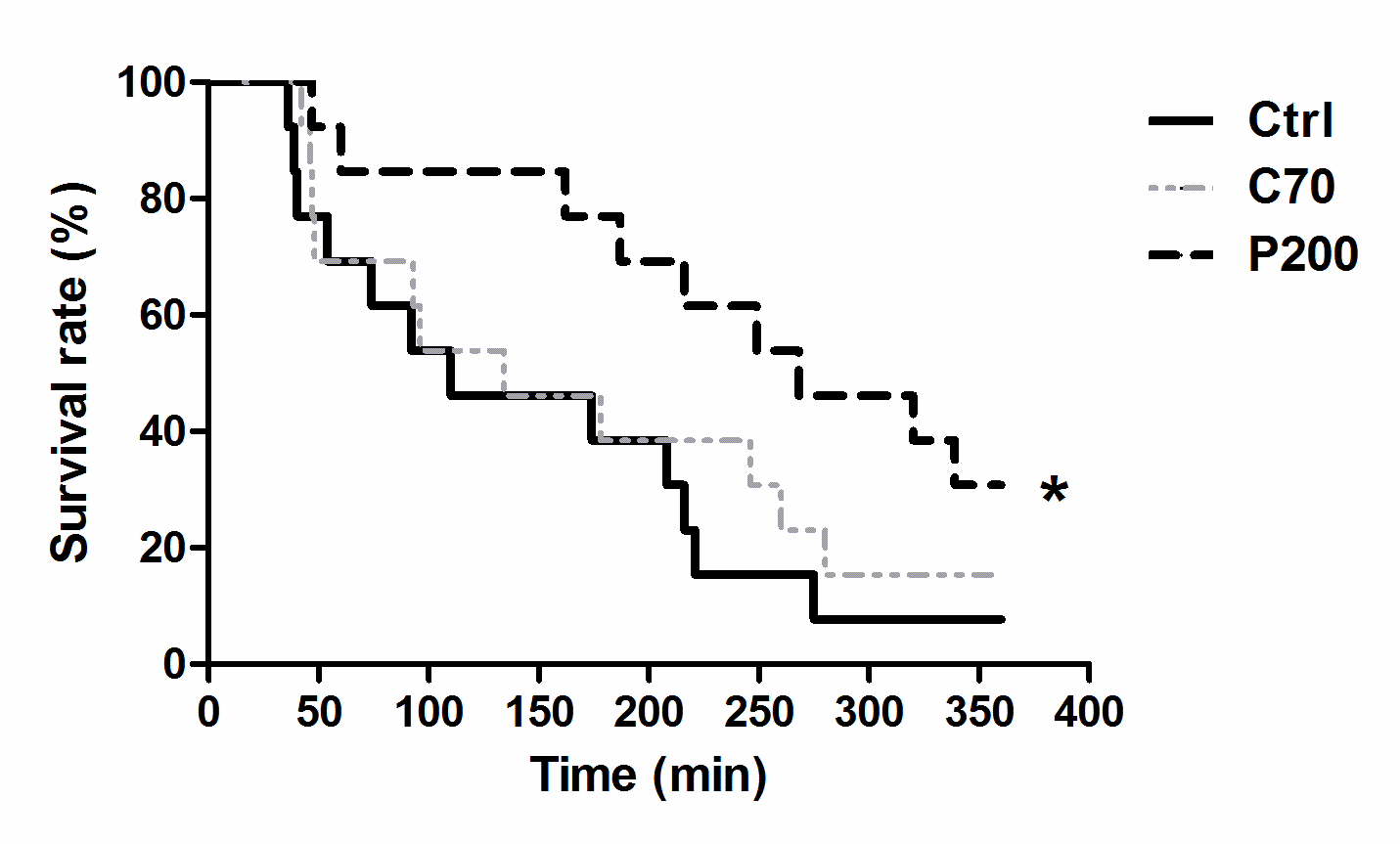

Supplement: S1 Fig — Lines for each group are defined as survival rate over time. The 50% survival time was 100, 120 and 280 min in Ctrl, C70 and P200 group, respectively. For group definition and symbols see Fig 4. * P<0.05 vs. Ctrl in log rank (Mantel-Cox) test, n = 13 in each group. (TIF) [file pone.0230229.s004.tif]
