# Supplementary material for: In vitro characterization and in vivo comparison of the pulmonary outcomes of Poractant alfa and Calsurf in ventilated preterm rabbits
Source: PLoS One. 2020 Mar 13;15(3):e0230229. doi: 10.1371/journal.pone.0230229 (PMC7069639; doi:10.1371/journal.pone.0230229)
Supplement: Attachment — Submitted filename: Response to Reviewers-1.docx [file pone.0230229.s005.docx]

Authors reply to each query (Q) and additional changes.

Journal Requirements:

1. When submitting your revision, we need you to address these additional requirements.

Please ensure that your manuscript meets PLOS ONE's style requirements, including those for file naming. The PLOS ONE style templates can be found at
http://www.journals.plos.org/plosone/s/file?id=wjVg/PLOSOne_formatting_sample_main_body.pdf and http://www.journals.plos.org/plosone/s/file?id=ba62/PLOSOne_formatting_sample_title_authors_affiliations.pdf

**Response:** We thank the editors for the instructions. We hope that our manuscripts meet the specified requirements.

2. At this time, we request that you  please report additional details in your Methods section regarding animal care, as per our editorial guidelines: 1) Please provide details of animal welfare for the does (e.g., shelter, food, water, environmental enrichment); 2) Please confirm that the pups were anaesthetised throughout the study and never gained consciousness; Please also clarify whether the does were anaesthetised when they were euthanized and 3) Please provide the source of the porcine lungs used in this study to isolate Poractant alfa. If the pigs were euthanized specifically for this study, please include the method of euthanasia. If they were obtained from a slaughterhouse, please provide the name, location, and accreditation. Thank you for your attention to these requests.

**Response:** 1) All does were housed until the twenty-sixth day of gestation under standard conditions of the animal center and were transported to the experiment site one day prior to the experiments with full shelter, food and water. Related contents are added to Method part, page 8, line 176-178, of R1-marked. 2) All does and pups were anaesthetized and never gained consciousness throughout the study. Does were sacrificed by overdose of potassium. Pups were euthanized by intracranial injection of 0.5 mL 2% lidocaine. Related content are add to Method part, page 8, line 187-189 and page 9, line 215-216, of R1-marked. 3) *Poractant alfa* was purchased by the Research and Development Department of CHIESI Farmaceutici, from the Sales Department of the same company. Therefore, all *Poractant alfa* vials used in this study were produced in compliance with all European regulations concerning the development of pharmaceutical products. Technically, all *Poractant alfa* vials are from a commercial source and no pigs were specifically euthanized for this study.

3. We note that you have included the phrase “data not shown” in your manuscript. Unfortunately, this does not meet our data sharing requirements. PLOS does not permit references to inaccessible data. We require that authors provide all relevant data within the paper, Supporting Information files, or in an acceptable, public repository. Please add a citation to support this phrase or upload the data that corresponds with these findings to a stable repository (such as Figshare or Dryad) and provide and URLs, DOIs, or accession numbers that may be used to access these data. Or, if the data are not a core part of the research being presented in your study, we ask that you remove the phrase that refers to these data.

**Response:** S1 Fig is added to Supporting Information to show the survival rate of groups over the 360 min observation period (also see response in Q8), and the phrase has been removed in R1.

4. Thank you for stating the following in the Financial Disclosure section:

The study was supported by Chiesi Farmaceutici S.p.A. The company contributed to the study design but had no influence in the performance, analysis, and interpretation of experimental data and in writing the manuscript. YD is a recipient of research grants from the National Natural Science Foundation (No. 81501288) and Shanghai Municipal Commission of Health (Project Young Physician Investigator).

We note that you received funding from a commercial source: Chiesi Farmaceutici S.p.A.

Please provide an amended Competing Interests Statement that explicitly states this commercial funder, along with any other [relevant declarations](http://www.plosone.org/static/competing.action) relating to employment, consultancy, patents, products in development, marketed products, etc.

Within this Competing Interests Statement, please confirm that this does not alter your adherence to all PLOS ONE policies on sharing data and materials by including the following statement: "This does not alter our adherence to PLOS ONE policies on sharing data and materials.” (as detailed online in our guide for authors <http://journals.plos.org/plosone/s/competing-interests>).  If there are restrictions on sharing of data and/or materials, please state these. Please note that we cannot proceed with consideration of your article until this information has been declared.

Please include your amended Competing Interests Statement within your cover letter. We will change the online submission form on your behalf.

Please know it is PLOS ONE policy for corresponding authors to declare, on behalf of all authors, all potential competing interests for the purposes of transparency. PLOS defines a competing interest as anything that interferes with, or could reasonably be perceived as interfering with, the full and objective presentation, peer review, editorial decision-making, or publication of research or non-research articles submitted to one of the journals. Competing interests can be financial or non-financial, professional, or personal. Competing interests can arise in relationship to an organization or another person. Please follow this link to our website for more details on competing interests: <http://journals.plos.org/plosone/s/competing-interests>

**Response:** We have extended the Competing Interests Statement as follows, and is added to cover letter.

The study was supported by Chiesi Farmaceutici S.p.A, which owns the marketing rights for *Poractant alfa*. The company contributed to the study design but had neither influence on the performance, analysis, and interpretation of experimental data nor in writing the manuscript. BP, SC, and FS are employees of Chiesi Farmaceutici S.p.A. XM served as consultant for Chiesi in this study. YD is a recipient of research grants from the National Natural Science Foundation (No. 81501288) and Shanghai Municipal Commission of Health (Project Young Physician Investigator). This does not alter our adherence to PLOS ONE policies on sharing data and materials.

Reviewer reports:

Reviewer #1:**Q1** The tables and figures were imbedded in the manuscript instead of at the end (out of order), and the figure legends don't tell a story of what it is they are trying to show. The take home message of each figure is not clear.

**Response:** We thank for the reviewer’s comments. We submitted the tables and figures according to the PLOS requirements, in which the tables and figure legends were embedded in the manuscript and the figures were submitted separately as TIFF forms. The figure legends have been rephrased for your reading convenience.

**Q2** The language and labeling is confusing- for example- C200 corresponds to poractant 200 mg/kg and K200 is calsurf. why would calsurf start with the K and not the C?

**Response:** We thank for the reviewer’s comments and feel sorry for the confusion. Reviewer 4 also mentioned this problem in Q25. Considering the generic nomenclature and to keep coincidence, the abbreviations of experimental groups are modified into C70, C100, C200 for 70, 100, 200 mg/kg *Calsurf* and P100, P200 for 100, 200 mg/kg *Poractant alfa* in R1.

**Q3** Also, the numbers in the tables don't always correspond with the results section. Relevance of results is not well explained.

**Response:** We thank for the comments and rephrased results related to tables and figures in R1.

**Q4** Statistics are lacking- for example table 1.

**Response:** We have analyzed and determined the surface roughness of *Poractant alfa* and *Calsurf*, which is a parameter representative of the microstructural complexity of the surfactant. We performed an ANOVA and then a t-test between *Poractant alfa* and *Calsurf* and both tests revealed a significant difference between preparations, showing a more complex three-dimensional structure of *Poractant alfa*.

In Table 1, we have completed the descriptive statistics by adding the standard deviation of the measurements for each class of phospholipids in the new version of the manuscript. For PL classes less represented in the surfactants, our NP HPLC-MS methods showed approximately a 20-30 % of difference in the quantitative estimation. This was in part due to the presence of some single species with intensities near the quantitative detection limit, and in part to the fact that data were acquired in full scan mode, by switching the two polarities, using an ion trap mass spectrometer. This approach, in our opinion, was adequate for the target of this work, a general comparison of the two surfactants.

Reviewer #2: With interest, I read the manuscript by Bo Sun et al about the characterization and comparison of two types of surfactant in preterm rabbits. The authors performed an impressive and complete vivo and vitro investigation. The study is well performed, well described and interpretable by readers with limited background in animal and in vitro studies. The study showed a favourable outcome for poractant. This study, although more detailed and done in animals, is one of many trying to identify a difference between the different surfactant products. Studies done in preterm infants have shown contradictory results, although generally in favour of poractant. Reviewing the literature, it is remarkable how much of the studies, as is this one, have been sponsored by surfactant manufacturers. It is my opinion that the surfactant research needs more independent studies. **Q5** The study is a head to head experiment, eventually using 60 rabbit pups in several experiments. The study was approved by the medical ethics counsel and suffering for the animals was minimised. Throughout the manuscript it remains somewhat unclear which rabbit underwent which investigation(s).

**Response:** We feel sorry for not having clarified enough the *in vivo* experimental design. Actually, in total, two *in vivo* sessions of experiments were performed: one was standardized PIP loop ventilation experiment, and the other, standardized V_T_ ventilation experiment.

In the PIP loop session, 60 rabbit pups were randomly allocated into six ventilation groups, namely P200 (*Poractant alfa* 200 mg/kg), P100 (*Poractant alfa* 100 mg/kg), C200 (*Calsurf* 200 mg/kg), C100 (*Calsurf* 100 mg/kg), C70 (*Calsurf* 70 mg/kg) and Ctrl (shame air) groups in this revised manuscript (n=10 in each group).

In the standardized V_T_ session, in addition to the former six groups (n=25 in each group in this session), we added one non-ventilated group (C_0_), which served as an additional control group (n=9) for biochemical analysis of broncho-alveolar lavage and lung tissue samples.

Some questions and suggestions remain,
**Q6** How is it possible that Calsurf is marketed without any preclinical or clinical studies? For bovine or calf derived surfactant there is enough evidence and clinical studies, how does Calsurf relate to these products?

**Response:** We appreciate this comment, which raised a relevant concern. We have conducted an additional search of articles on PubMed with the keywords “Calsurf” or “Kelisu”. As a result, we find only one additional research published: Rong Z, et al. A multicetered randomized study on early versus rescue calsurf administration for the treatment of respiratory distress syndrome in preterm infants. Am J Perinatol. 2019 Feb 4. doi:10.1055/s-0039-1678530. This publication has been added to Introduction part as reference [17]. Neither surface properties nor comparisons to other surfactants have been published yet to the best of our knowledge. We can only know Kelisu has similar formulation in terms of phospholipid concentration (roughly about 30 mg/ml) as other bovine derived surfactants (reviewed in [18] in revised manuscript). Related contents are added or rephrased in page 4, line 82-88, in R1-marked.

**Q7** Line 351-352 PEEP or no PEEP, If I look at figure 3, I see, possibly not significant, a positive effect of PEEP on the Cdyn. The positive effect of C200 is amplified by PEEP. Would have liked to see higher PEEP levels, although not investigated in this study.
When looking further at figure 3, it strikes me that K100 outperforms C100 and that C100 is so much ‘worse’ than C200. Any thoughts on that?

**Response:** We thank for the comment. To clarify this issue, we replaced the acronyms of experimental groups in the revised manuscript (modified according to comments by Reviewer 1 and 4 in Q2 and Q25): P200 (*Poractant alfa* 200 mg/kg), P100 (*Poractant alfa* 100 mg/kg), C200 (*Calsurf* 200 mg/kg), C100 (*Calsurf* 100 mg/kg), C70 (*Calsurf* 70 mg/kg) and Ctrl (shame air) groups.

PEEP indeed had a positive effect on C_dyn_, not only in P200 group but also in all groups including Ctrl group, with no statistic significance. However, the effect of PEEP was not the aim of this study, so we didn’t highlight this point in the manuscript. Showing both results in Fig 3A and 3B was only to clarify the similar effects of surfactants both in dose and category way. Higher PEEP was not applied because of the higher requirements of ventilation instruments.

Considering deviations, C_dyn_ in C100 didn’t outperform P100, and the possible reason why P100 was so much “worse” than P200 may be related to the little fluid volume of exogenous surfactant, which was only 1.25 ml/kg in P100 group (about 0.03 ml per pup assuming birth weight was 27 g). The little fluid volume may have a negative effect on surfactant diffusion in premature lung.

**Q8** Line 365 – 377 This seems to be an irrelevant finding and does not support the use of Poractant. The control group was not treated with surfactant. It is well known that they do not survive. Also for Table 2. Of course there is a difference between the control group and the poractant group. But this was not the object of the investigation. Also for 473 – 474 and 554-555, the control group is not under investigation.

**Response:** We thank for the comments and queries. This protocol of mechanical ventilation, along with intermittent intraperitoneal injection of mixed solutions (Lidocaine+NaHCO_3_+Glucose), aiming at providing extra anaesthesia, energy and counterbalancing acidotic deterioration, ensured consistently the assessment of surfactant effects when the total length of ventilation may be 3-6 hours (Fig. 4 and S1 Fig. in R1). S1 Fig. is added in R1 to illustrate the survival length in the Ctrl, P200 and C70 groups. The survival length for the other three surfactant-treated groups (P100, C200 and C100) may be estimated thereupon. We applied a log-rank test as stated in the Methods (page 13, line 289) of the body text for comparison of the survival status across the groups. With this test statistic, the survival length in the surfactant-treated groups was compared step-wise in time interval with those in the control group which received identically standardized mechanical ventilation. This enabled estimation of either 50% survival (and death as reciprocal) at different time(s) or overall survival rate at a designated time over the whole ventilation period. Thus, it supports the notion that the length of survival is one of the major advantages in assessment of dose-effect of surfactant preparation.

**Q9** In figure 4 it is hard to discriminate between the different lines

**Response:** Thanks for your comments. Lines in figure 4 have been modified as P200, *Poractant alfa* 200 mg/kg, thick black dash line; P100, *Poractant alfa* 100 mg/kg, thick dark grey dash line; C200, *Calfsurf* 200 mg/kg, thin black dotted line; C100, *Calsurf* 100 mg/kg, thin grey dotted line; C70, *Calsurf* 70 mg/kg, thin light grey dotted line; Ctrl, shame air, black solid line.

**Q10** Reference 6 has a 2019 update

**Response:** We thank for the comments. Reference 6 has been updated.

Reviewer #3: This is a bench research work comparing two surfactant preparations with a multitude of methods. Practant alfa and Calsurf are compared by various in vitro tests and also in vivo in rabbit parts. Methods include the measurement of lipid composition, atomic force microscopy, Langmuir Blodgett analysis, and in vivo effects in premature rabbit pups. In the latter, compliance, survival time, and lung histology were measured. By all means, results point in the same direction, with both surfactants having typical effects, but poractant alfa function was superior.**Q11** In that respect, this reviewer cannot follow the author's conclusion that large clinical study comparing both surfactants might be warranted. Such a study might be unethical in view of the superior results found in all aspects with poractant alfa, because it is likely to expose a large number of infants to an inferior surfactant preparation. Equipoise, the prerequisite of randomization, can no longer be claimed after the described results have been found.

**Response:** We thank the reviewer for the comments. So far, no clinical trials have been conducted to compare the effects between *Poractant alfa* and *Calsurf* in preterm infants. Besides, the effects of *Poractant alfa* and other bovine or calf surfactants are not clear. Meta analysis in 2017 (reference [34] in R1) concluded *Poractant alfa* may be better, but could not tell whether it was related to animal source or initial dose (related discussion is added to page 24, line 540-541, of R1-marked). Accordingly, page 28, line 636-639, of R1-marked, the last sentences are modified or deleted.

**Q12** The abstract does not really reflect what was done in the paper, and what the results were. It may be misleading in referring only to Calsurf dosages of 70-100 mg per kilogram, whereas in fact, Calsurf was also tested at 200 mg per kg. Revising the abstract might be warranted.

**Response:** We thank for the comments. The arrangement and results regarding the comparison between the two surfactant preparations in abstract have been modified, as suggested.

**Q13** line 477: practice, not praxis

**Response:** Thanks, amended.

Reviewer #4: The authors of this manuscript present a very comprehensive in vitro and in vivo analysis of these two surfactant preparations. Upon revision, these data will be an important contribution to the exogenous surfactant literature.

Major Criticisms

**Q14** 1. The authors use matching doses of 100 mg/kg and 200 mg/kg of both surfactants but only 70 mg/kg of Calsurf. The authors need to explain in the manuscript why there is no Poractant alfa 70 mg/kg for comparison.

**Response:** We thank the reviewer for the comments. From the manufacturer information, *Poractant alfa* is licensed to 100-200 mg/kg and *Calsurf* is 40-100 (average 70) mg/kg. Considering the comparisons in both licensed dose and mg-to-mg ways, we set *Poractant alfa* in 100 and 200 mg/kg doses, *Calsurf* in 70, 100 and 200 mg/kg doses, so that 100 and 200 mg/kg of two surfactants were for head-to-head comparison and *Calsurf* 70 mg/kg was purposely added for licensed dose comparison with *Poractant alfa* 100 and 200 mg/kg. Considering that Poractant alfa will never be administered at doses below 100 mg/kg, we do not see the reason to add a *Poractant alfa* 70 mg/kg group for comparison.

**Q15** 2. For all experiments, the authors report statistical comparisons between experimental and control animals (which is important), but neglect to report statistical comparison between experimental groups (except for compliance (line 391) and lung injury score C200 vs K70 (line 456)). Despite this omission, they repeatedly state that one surfactant is superior to the other (lines 445, 467, 493, 553, 574, etc)

**Response:** We thank for the comments and have rephrased our results in *in vivo* studies. In Table 3, we report significance in lung injury scores between P200 (*Poractant alfa* 200 mg/kg) vs. other surfactant-treated groups in the Results, page 22, line 501-507, of R1-marked. We draw the statement that *Poractant alfa* was superior to *Calsurf* for two reasons: one was the direct comparison between the two surfactants, in which LIS was significantly lower in P200 group than in all other surfactant-treated groups (Table 3); the other was the indirect comparison for the two surfactants compared to the Ctrl group, in which only P200 group could reach the statistical significance in terms of C_dyn_ (PIP=20 and 15 cmH_2_O), survival time, W/D and CV [Vv] but no shown in the other surfactant-treated groups (Fig. 3, 4 and Table 2).

**Q16** 3. Methods (line 196) and associated Results/Fig 3 – There needs to be both a discussion and references to why the authors used no PEEP and a low PEEP such as 2-3 cm H2O. In part, this could be a reason why administration of surfactant did not statistically improve compliance (except for C200)(line 349, Fig 3) which is surprising. In figure 3, given the small SD for the control, it is surprising that there is no statistical difference. The authors may need to recheck their statistical analysis. For the figure 3 legend, it is not clear what “*”, “**”, and “#” refer to.

**Response:** We thank for the kind comments and feel sorry for the irrelevant presentation. For the Fig 3 legend, # refers to *P*<0.01 vs. all surfactant-treated groups; ** to *P*<0.01 and * to *P*<0.05 vs. Ctrl group. Accordingly, all surfactant-treated groups had a significantly higher C_dyn_ during the first 15 min and the last 5 min of the PIP loop experiment (when PIP=25 cmH_2_O). When lowering PIP to 20 and 15 cmH_2_O, only P200 group exerted significance compared to Ctrl group (*P*<0.01 when PIP=20 cmH_2_O, *P*<0.05 when PIP=15 cmH_2_O). The response to surfactant administration in terms of C_dyn_ was similar with or without PEEP=2-3cmH_2_O. The reason why the PIP loop was performed in both no- and low-PEEP was to verify whether there exists any differences of the surfactants response to PEEP. Related comments are also provided by Reviewer 1 in Q7. Two references [23, 24] and two sentences have been added in the Method part, page 9, line 217-218, and page 10, line 222, and Result part, page 17, line 379-381, and page 18, line 401-402, of R1-marked.

**Q17** 4. Methods (line 208) – For the prophylactic surfactant treatment, the authors report “A PIP ranging between 10-25 cmH2O was applied to generate a VT of 4-6 mL/kg body weight” (line 208). It is clear that pulmonary compliance changes quickly after administration of surfactant. During the 3 hour experiment, how often was the PIP titrated to maintain a goal VT of 4-6 mL/kg?

**Response:** We thank for the comments. During the first 30 min of ventilation, PIP was titrated in 3-5 minute interval, and subsequently, when the Cdyn (dynamic compliance of respiratory system) was relatively stable, PIP was adjusted at 10-15 minute interval. Related content is added to Method part of the body text, page 10, line 235-237, of R1-marked.

**Q18** 5. Figure 3 and Table S2 – There needs to be some discussion of why dynamic compliance was so different between the PIP ventilation experiment and the survival experiment. For example, at 15 minutes for C200, compliance was ~0.5 in figure 3A but was 0.28 in table S2. The authors seem to pick the data they prefer by stating in the discussion, “…was associated to significant improvement of Cdyn..” (line 531). This may be true for the data in Table S2 but not for the data in Figure 3.

**Response:** To clarify the differences of C_dyn_ levels (at 15 min, e.g.) in Fig 3 (as in the response to Q16) and S2 Table, different PIP was used in the PIP loop and the Vt standardized ventilation for survival. In the PIP loop ventilation, PIP=25-25-25-20-15-25 cmH_2_O, when PIP=25 cmH_2_O with or without PEEP, tidal volume could reach 8-10 ml/kg in P200 (*Poractant alfa* 200 mg/kg) group as a very good response while C_dyn_ reached ~0.5 ml/kg/cmH_2_O. In the survival experiment with standardized V_T_ ventilation, PIP was adjusted in a range of 10-20 cmH_2_O, with PEEP at 2-3 cmH_2_O, to achieve 4-6 ml/kg of V_T_. Accordingly, C_dyn_ in the two different ventilation modes were not comparable. One sentence is added to Discussion, page 27, line 595, of R1-marked to clarify the issue.

**Q19** 6. Results – For the AFM, Langmuir Blodgett and lipid analysis, why is there no quantitative comparison or statistical analysis? How do we know if the samples are statistically different? For the lipid analysis, why were only 4 samples tested for Calsurf but 8 samples tested for Poractant alfa and why are standard deviations omitted?

**Response:** We have analyzed and determined the surface roughness of *Poractant alfa* and *Calsurf*, which is a parameter representative of the microstructural complexity of the surfactant. We performed an ANOVA and then a t-test comparing *Poractant alfa* and *Calsurf*. Both tests revealed a significant difference between preparations, showing a more complex three-dimensional structure of *Poractant alfa*. For this application, we considered the Langmuir Blodgett results as qualitative data and we did not perform any statistical analysis.

In Table 1, we have completed the descriptive statistics by adding the standard deviation of the measurements for each class of phospholipids in the new version of the manuscript. For PL classes less represented in the surfactants, our NP HPLC-MS methods showed approximately a 20-30 % of difference in the quantitative estimation. This was in part due to the presence of some single species with intensities near the quantitative detection limit, and in part to the fact that data were acquired in full scan mode, by switching the two polarities, using an ion trap mass spectrometer. This approach, in our opinion, was adequate for the target of this work, a general comparison of the two surfactants. Unfortunately, due to the limited *Calsurf* availability (e.g. not in Europe, only in China) we could only perform 4 LCMS runs with this particular preparation.

**Q20** 7. Results (line 373) – for the survival analysis, the authors mention a 360 minute ventilation experiment but don’t include these data. These data should be included and the protocol needs to be described in the methods section.

**Response:** We thank for comments. The additional experiment was conducted in the same standardized V_T_ ventilation protocol except for the observation time for 360 min in P200 (*Poractant alfa* 200 mg/kg), C70 (*Calsurf* 70 mg/kg) and Ctrl (sham air) groups. We confirmed that the 50% survival time of Ctrl and C70 group was around 100 and 130 min and that of P200 group could reach 280 min. These results have been submitted as S1 Fig in Supporting information. See also on response to Q8. Related content is added to Results part, pages 18-19, lines 415-421, of R1-marked.

**Q21** 8. Discussion – the authors should add speculation for why the lipid species are so different in Table 1, yet when given to rabbits, they are not very different in lung homogenate or BAL fluid (Figure 5).

**Response:** We thank for the comments. There may exist two reasons for this speculation. For the first, although lipid species detected in Table 1 were abundant including cholesterol and several kinds of phospholipids, the percentage of saturated PL were both around 50%, which was consistent with Fig 5F though lacking significant differences among groups. For the second, as with the metabolism of exogenous surfactants in premature lungs, the surfactants were taken up by alveolar epithelial cells and reutilized as ingredients. A speculation is already presented in the Discussion, pages 25-26, lines 565-569, of R1-marked, regarding the biophysical behaviour of phospholipid composition of the two preparations. The total phospholipids (TPL) and disaturated phosphatidycholine (DSPC) detected in the experiment were those in the alveolar and intracellular pools, not identical to those from exogenous surfactant itself. In other words, these phospholipids were mixed up with endogenous phospholipids in each compartment pool.

**Q22** 9. Discussion – the authors should add speculation to reconcile the lack difference in compliance between surfactants yet improved survival. What is the mechanism?

**Response:** We thank for the comments. The facts that increment of surfactant dosage (by phospholipids) may be responsible for the prolonged survival time with association of stabilized compliance. There were not significant differences in C_dyn_ among surfactant-treated groups in standardized V_T_ ventilation. We speculate that the relatively low V_T_ should have exerted protective role in the difference of LIS in Table 3. A low dose of surfactant may be associated with its uneven distribution in the premature lungs provocative of alveolar and small airway damage and pneumothorax, leading to early death in the control and low-dose surfactant-treated groups. This is added in the Discussion, page 25, line 552-557, of R1-marked.

**Q23** 10. Discussion (lines 487, 551) - The authors point out the importance of the fluid volumes of the instilled surfactant, yet don’t include this data in the methods or results. For each of the experiments presented, what volumes of surfactant were instilled?

**Response:** We thank for the kind comments. The fluid volume for P200 (Poractant alfa 200 mg/kg), P100 (Poractant alfa 100 mg/kg), C200 (Calsurf 200 mg/kg), C100 (Calsurf 100 mg/kg) and C70 (Calsurf 70 mg/kg) groups were 2.50, 1.25, 5.71, 2.86 and 2.00 ml/kg respectively. Related contents have been added in Methods, page 9, line 205-208, of R1-marked.

Minor Criticisms

**Q24** 1. It is noted in the PLOS One competing interests statement that four of the authors are employees of Chiesi including Davide Amidani. For Davide Amidani, this needs to be noted in the manuscript itself for readers to be aware of (line 5).

**Response:** We have corrected the authors’ affiliations and we have extended the competing interest statement as:

The study was supported by Chiesi Farmaceutici S.p.A, which owns the marketing rights for *Poractant alfa*. The company contributed to the study design but had neither influence on the performance, analysis, and interpretation of experimental data nor in writing the manuscript. BP, SC, and FS are employees of Chiesi Farmaceutici S.p.A. XM served as consultant for Chiesi in this study. YD is a recipient of research grants from the National Natural Science Foundation (No. 81501288) and Shanghai Municipal Commission of Health (Project Young Physician Investigator). This does not alter our adherence to PLOS ONE policies on sharing data and materials.

**Q25** 2. Throughout the manuscript, the use of abbreviations based on the trade names is confusing. Consistent with the generic nomenclature, the abbreviations should be Calsurf (C70, C100, C200) and Poractant alfa (P100, P200). Trade names should be omitted from the manuscript except maybe once in the methods section.

**Response:** Thanks for the comments. The experimental groups and trade names of surfactants have been replaced accordingly in the revised manuscript.

**Q26** 3. For the pathology analysis (line 246), the authors need to specify whether or not the pathologist was blinded or not to the experimental group.

**Response:** We thank for the comments. The pathologist was blinded to the experimental design. Related content is added to Methods, page 12, line 274, of R1-marked.

**Q27** 4. Results (line 410) – The authors state “…suggesting DSPC/TP to be a sensitive indicator for lung injury protection.” Surfactants have phospholipids and surfactants can help, in some ways, to prevent lung injury. This does not mean, nor does the data provided, support that this ratio is a sensitive indicator. This statement should be omitted.

**Response:** We thank for the comments. This statement has been omitted in the revised manuscript.

**Q28** 5. Results (line 437) – “V_V_ accounts for improved alveolar expansion at the end of expiration and is a good estimate to assess the maintenance of functional residual capacity (FRC)” – a reference should be provided to support this statement.

**Response:** Reference [31] has been provided in page 22, line 490, of R1-marked.

**Q29** 6. Results (line 283) – DPPC is spelled wrong as “pipalmitoylphosphatidylcholine”
**Response:** It has been changed.

**Q30** 7. Results (line 409) – “folds” should be “fold”

**Response:** It has been changed.

**Q31** 8. Discussion (line 477) – While the term “praxis” may technically be correct, the term “practice” is more understood

**Response:** It has been changed.

**Other changes in R1 not related to reviewers’ and editors’ comments:**

Minor changes about language for more readable. Reference [25-26] is added for the original method in phospholipid analysis.
