# Supplementary material for: In vitro characterization and in vivo comparison of the pulmonary outcomes of Poractant alfa and Calsurf in ventilated preterm rabbits
Source: PLoS One. 2020 Mar 13;15(3):e0230229. doi: 10.1371/journal.pone.0230229 (PMC7069639; doi:10.1371/journal.pone.0230229)
Supplement: Attachment — Submitted filename: Response to Reviewers-R2.docx [file pone.0230229.s006.docx]

Authors reply to the remaining query (Q) and additional changes.

Reviewer #1: The authors have addressed many of the reviewers comments. I believe there are a few more adjustments that are necessary before publication.

1. In the body of the abstract, the authors need to include in the methods section that the company Chiesi Farmaceutici S.p.A. contributed to the study design.

**Response:** We take into consideration the Reviewer’s comment. According to our previous experience, in the final layout of PLOS ONE, the conflict of interest is declared in the front page of the article, just below the abstract. We have already declared our conflict of interest during the submission process, which will be accordingly displayed in the front page, provided that this work is accepted.

That said, if this would not be enough, we are willing to include the sentence suggested by Reviewer#1 in the abstract if the editor finds it more appropriate.

2. Q14 and Q15 revision response should be added to the discussion of the manuscript.

**Response:** Regarding Q14, we have included the following sentence in the discussion section explaining why we did not include a group of 70 mg/kg of *Poractant alfa* (in line 530-535, page 25, of R2-marked):

“*To carry out a sound head-to-head scientific comparison between surfactants, we included additional experimental groups of Calsurf-treated rabbits with dosages of 100 and 200 mg/kg, which would match the licensed doses of Poractant alfa. Considering that Poractant alfa is not licensed to be administered at doses below 100 mg/kg, we did not include a group of animals treated with Poractant alfa at a dose of 70 mg/kg*”.

**Regarding Q15, we would like to clarify that, from the beggining, we always compared statistically all groups between them (including surfactant-treated groups) with the statistical methods reported in the “statistical analysis” section (line 287-288, page 13, of R2-marked).** However, we only reported significant differences in the figures if P<0.05. For instance, in the ventilation loop and in the mortality curve, there were no significant differences between surfactant-treated groups; only between P200 and untreated but ventilated controls. To make this point clear for the readers, we have included new sentences in the results and discussion sections clarifying in which cases no significant differences were observed between surfactant-treated groups and also sentences indicating when the significant differences were observed between P200 and the control group:

Line 391-392, page 18, of R2-marked: *“Irrespective of the surfactant preparation or the administered dose, no significant differences were found if surfactant-treated groups were compared”.*

Line 418-419, page 20, of R2-marked: **“***However, no significant differences were detected after comparing surfactant-treated groups”.*

Line 442-443, page 21, of R2-marked: “*However, no significant difference was detected between surfactant groups with different doses”.*

Line 537-539, page 25, of R2-marked: *“Nevertheless, a significant reduction of RDS-associated mortality compared to untreated but ventilated controls was only achieved after treatment with 200 mg/kg of Poractant alfa”.*

Line 635-638, page 29, of R2-marked: “*We therefore suspect that the significant differences observed between Poractant alfa administered at a dose of 200 mg/kg and untreated control animals in the PIP loop experiments…”.*

Reviewer #2: I would like to thank the authors for their answers to my questions and wish them the best with their investigations.

**Response:** We thank the reviewer for the criticism, which has improved the quality of the manuscript.

Reviewer #4: The authors have addressed many, but not all, of my previous concerns. Although the original manuscript was written in acceptable English, the revision has many statements that are not intelligible and not written in standard English.

**Response:** We thank the reviewer for the advice. We have revised the manuscript and shortened and English-proofed some sentences to make the statements clearer and more comprehensible for the readers.

Remaining concerns:

Q15. The authors still did not perform a statistical analysis to determine if the two surfactants are different in Figures 3 and 4 and Table 2. Without this, it is not accurate to repeatedly state that one surfactant is superior to the other. An indirect comparison for the two surfactants compared to the control group is not an acceptable substitute.

**Response:** Regarding Q15, we refer to the response given to the 2^nd^ concern of Reviewer #1. In the present version of the manuscript, **we clearly state that in figures 3 and 4 and table 2 statistical analysis between all groups has been performed,** and there are not significant differences between surfactant-treated groups and that the significant differences were only detected between P200 and untreated control animals.

This has also been amended in the discussion section.

Q19. The authors still did not perform a statistical analysis for the Langmuir Blodgett analysis (Figure 2) or for the Lipid analysis (Table 1). Both of these appear to very quantitative experiments. In the current manuscript, there is no way to know if there is a proven difference between surfactants for the experiments in Figure 2 and Table 1. For example, in the Langmuir Blodgett analysis, it is not valid to comment on any difference in plateau surface pressure or peak compressibility without a statistical test.

**Response:** We agree with Reviewer#4 that these techniques yield quantitative data. However, we believe that the most interesting observations from Table 1 and Figure 2, and also Figure 1 (AFM) are typically qualitative (as applied already many times in the available literatures. See below additional references).

The LC-MS data revealed qualitative differences between both formulations and in particular the absence of plasmalogens in *Calsurf* and the absence of cholesterol in *Poractant alfa*. It must be noted that the analysis was performed using 1 mL of native surfactant preparations, which are formulated at concentrations of 35 mg/mL (Calsurf) and 80 mg/mL (Poractant alfa), which accounts for a 3-, 2-, 10-, and 3-fold higher PC, DPPC, PE and PI content in *Poractant alfa* compared to *Calsurf*. We believe that these data already show evident differences between both surfactants.

Regarding the Langmuir Blodgett analysis, we have measured the value of the compressed area reached by *Poractant alfa* and *Calsurf* at a surface pressure of 68 mN/m. We have chosen 68 mN/m because both surfactants reached this very high value of pressure in these trials.

Under these analytical conditions, the comparison between surfactants did not reach statistical significance (P>0.05), being 14.6 ± 0.35 Å^2^/molecule for *Poractant alfa* and 13.8 ± 0.76 Å^2^/molecule *for Calsurf*. We have included this comparison in the present version of the manuscript (line 376-378, page 18, of R2-marked).

That said, we believe that the shape of the isotherms is qualitative a much more relevant information and it indicates some differences between both preparations which are discussed in the manuscript. This is the reason why we included just qualitative observations in previous versions of the manuscript.

We include below some references in which LB experiments have been used to qualitatively characterize composition and activity of pulmonary surfactants:

1. H Zhang, Q Fan, YE Wang, CR Neal, YY Zuo. Comparative study of clinical pulmonary surfactants using atomic force microscopy. Biochimica et Biophysica Acta. 2011; 1808: 1832–1842.

2. D Lukovic, A Cruz, A Gonzalez-Horta, A Almlen, T Curstedt, I Mingarro, J Pérez-Gil. Interfacial behavior of recombinant forms of human pulmonary surfactant protein SP-C Langmuir. 2012; 28: 7811−7825.

3. DM Schenck, J Fiegel. Tensiometric and phase domain behavior of lung surfactant on mucus-like viscoelastic hydrogels ACS. Appl. Mater. Interfaces. 2016; 8: 5917−5928.

4. RP Valle, CL Huang, JSC Loo, YY Zuo. Increasing hydrophobicity of nanoparticles intensifies lung surfactant film inhibition and particle retention ACS. Sustainable Chem. Eng., 2014; 2: 1574−1580.

Q25. The Trade names continue to be in the article title. These should be removed.

**Response:** We understand the concern of the reviewer. Following the Reviewer’s advice, we have removed the trade names of the products from the title.

Q28. The authors added reference 31 (Ennema JJ et al) to support the statement “Vv…is a good estimate to assess the maintenance of functional residual capacity (FRC).” The referenced publication makes no mention of FRC and therefore does not support a previously proven correlation between Vv and FRC.

**Response:** What we meant in this sentence is that Vv accounts for improved alveolar expansion at the end of expiration, as well FRC is essential to prevent alveoli collapse at the end of expiration. The method of Vv in this animal model was developed in late 1970’s and 1980’s by Dr. Bengt Robertson and his colleagues adopting Weibel’s theorem of stereometric measurement of lung volume to estimate expansion magnitude of alveoli (a gas volume approximate to FRC, which was further verified by nitrogen wash-out methods in clinical settings for human neonates in 1990’s). Different from the previous experience, the animals in our study were succeeded to be ventilated with 2-3 cmH_2_O PEEP in contrast to 5-6 cmH_2_O in clinical settings for RDS, which should have achieved a better, though still lower, level of FRC. Furthermore, the method of perfusion fixation of the animal lungs in this study may not ensure the expanded alveoli to be commensurable to FRC. Therefore, Vv should at best approximate to residual volume (RV), a part of FRC, in these premature lungs, in the presence or absence of exogenous surfactants.

Considering these, we additionally think that this parameter can be a good estimate to assess the manteinance of FRC. To clarify this concern, we have modified the position of reference 31 as follows: (Line 492-494, page 23, of R2-marked)

*“V_V_ accounts for improved alveolar expansion at the end of expiration[31] and is a good estimation of residual volume reflecting the maintenance of functional residual capacity (FRC) under various treatment”.*

**Additional changes in R2:**

We included two short sentences and reference [20] in the methods section of the Langmuir Blodgett measurements of the main body text (Line 159-163, page 8, of R2-marked) to better understand the relationship of surface tension and surface pressure.
